# The Factors Influencing Chinese University Teachers’ Intentions for Using the Micro-Lecture in the Post COVID-19 Era

**DOI:** 10.3390/ijerph192214887

**Published:** 2022-11-12

**Authors:** Qihui Xie

**Affiliations:** Department of Public Administration, School of Law and Humanities, China University of Mining and Technology (Beijing), Beijing 100083, China; xieqihui@cumtb.edu.cn

**Keywords:** post COVID-19 era, university teachers, micro-lecture usage intention, TAM, UTAUT

## Abstract

The COVID-19 pandemic has produced a far-reaching influence on higher education and the teaching activities of teachers in Chinese universities. The intentions of teachers in universities for using the micro-lecture, one of the educational informationization products, and the influencing factors of the intentions for using micro-lectures, are changing in the post COVID-19 era. This paper, based on the Technical Acceptance Model (TAM) and the Unified Theory of Acceptance and Use of Technology (UTAUT), constructed the research hypotheses for the influence factors of micro-lecture usage intentions of teachers in universities in the post COVID-19 era, and made corresponding verifications through the Structural Equation Model (SEM). As shown by the results therefrom: (1) the micro-lecture usage experience before the outbreak of the COVID-19 pandemic significantly affected the usage intentions of teachers in universities; (2) the perceived usefulness influenced the usage intention directly, but the perceived ease of use did not directly produce influence; (3) policy impact had no significant influence on the perceived usefulness and the perceived ease of use of university teachers for micro-lecture use; (4) social relations and personal innovativeness have significant impacts on perceived usefulness, teaching objectives and micro-lecture characteristics have significant impacts on the perceived ease of use. In this paper, suggestions and opinions on popularizing micro-lecture usage in the post COVID-19 era were put forward on the basis of research conclusions therein.

## 1. Introduction

Since 2020, the COVID-19 pandemic swept across the globe and has changed the lives of people, and also higher education. As it developed, China was almost making the largest scale of online educational experiments in the world’s history [1], so traditional higher education suffered a severe impact, the means, contents and evaluations of higher education were changed. As the COVID-19 pandemic is tending towards stability, the post COVID-19 era is gradually arriving; the post COVID-19 era means a period in which the influences of the COVID-19 pandemic are tending to disappear, but have not disappeared completely [2]. The influences of the COVID-19 pandemic can be understood from two aspects; firstly, the native influences of the virus have not disappeared, and secondly, the influences of the COVID-19 pandemic on society, economy, culture and education are still ongoing [3,4]. In this paper, the focus is the exploration of the second influence in the post COVID-19 era, especially the influence of the COVID-19 pandemic on higher education. The COVID-19 pandemic has dual influences on higher education, on the one hand, it adds to the difficulties of higher education, for example almost all offline courses throughout the world were suspended [5]; on the other one hand, it brings opportunities for the development of higher education in the Internet era, promotes the development of online education in a large scale, and simultaneously, improves the level of educational informationization in its outbreak. The combination of education and informationization meets the inevitable trend of the internet era, but also, it is the development direction of future education. In the development trend of China’s internet, surfing the Internet by cell phone or other mobile terminals is becoming a trend. By June 2022, there were 1.051 trillion netizens in China, including 1.047 trillion cell phone netizens, where 99.6% netizens surfed the internet by cell phones. However, usage rates of the desktop computer, notebook computer and IPAD, respectively, declined [6]. Traditional online study is turning toward cell phone online study, the micro-lecture, a product aimed at meeting the development demand of surfing the Internet by cell phone, is attracting more and more attention from teachers in the field of Chinese higher education.

However, there is fuzzy definition of the micro-lecture in the present research, some scholars thought the micro-lecture was a kind of teaching resource [7,8,9,10] or a kind of teaching process [11]; also, some others thought the micro-lecture was, in particular, a network course [12]. On the basis of previous research, the study in this paper figured out and analyzed the origin and the connotations of the micro-lecture concept, and was expected to finalize the definition of the micro-lecture and increase the literature relating to the analysis of the concept of micro-lecture. Additionally, the present quantitative research on the usage intentions of micro-lectures was carried out mostly from the perspective of students [13], but less from the perspective of teachers; moreover, the variables about the COVID-19 pandemic were seldom to be incorporated into research models. In this paper, usage experiences before and after the COVID-19 pandemic were incorporated into the quantitative research model, and the study was carried out from the perspective of university teachers.

This paper, based on the literature review of the micro-lecture, TAM and UTAUT, constructed the research model of factors influencing university teachers’ intentions for using micro-lecture; incorporated the control variables of the usage history before COVID-19 pandemic, the usage experience in the course of COVID-19 pandemic, sex, age, discipline and tile into the model; and put forward to the research hypotheses: (1) perceived usefulness and perceived ease of use might have a positive influence on micro-lecture usage intentions; (2) perceived ease of use can have a positive influence on perceived usefulness; (3) external environments such as social relations, policy impact, teaching objectives, micro-lecture characteristics and personal innovativeness might have positive influence on perceived usefulness and perceived ease of use. Through the way of accidental sampling, research questionnaires were released to eight universities located along the Xueyuan Road, Haidian District, Beijing City, namely, the area in which China’s typical high-level universities are gathered. In the paper, the analysis of questionnaire data and the validation on research hypotheses were made by using the SEM. The research conclusions in this paper can provide opinions and suggestions for promoting teachers in universities for developing micro-lecture teaching resources, using micro-lecture education style and reforming and innovating the styles and methods of higher education.

## 2. Literature Review

### 2.1. Micro-Lecture

#### 2.1.1. Origin and Connotations of Micro-Lectures

1. Origin

Presently, what is generally recognized by scholars is the concept of the micro-lecture that was put forward by David Penrose from San Juan College in New Mexico, USA in 2008 [14,15], he developed the “one-minute micro video” and called it the “micro-lecture”, namely the teaching of content in 1~3 min that can lead students to use the powerful tool of the internet to realize online study and mobile study according to the available resources and activities, and thereby acquire the knowledge that he/she is eager to learn. According to David Penrose, the micro-lecture is a kind of “knowledge pulse”, and it can obtain the same teaching result as that of traditional and long-time school teaching, with the corresponding support of homework and discussion. It means that the micro-lecture can not only be used for producing science popularization education, but also for regular classroom teaching. However, before the concept of the micro-lecture was officially put forward, education departments and educational institutions in various countries had attempted to put forward similar concepts, see Table 1 as follows. There are different names, such as micro-course, micro-teaching, micro-video, etc., but their common point of interest is to help students to use their fragmented time to study, so the micro-lecture was formed on the basis of those concepts therein, and was finally put forward by David Penrose.

2. Connotations

Presently, the definition of the micro-lecture concept is not unified in academic circles. It is right because the concept of the micro-lecture has undergone three development phases from its emergence to the present, and scholars have different understandings of the micro-lecture. Such three development phases are known as the teaching resource phase, teaching process and online course. Table 2 summarizes the forms, use and contents of three phases.

The teaching resource phase is the initial research phase of the concept of the micro-lecture. In this phase, scholars thought the connotation of the micro-lecture was a kind of teaching resource, it could be embodied by a short teaching video, teaching courseware, materials or exercises. The characteristics of such resources are the short and snappy content focusing on describing a certain knowledge point and teaching process [7,8,9]. For example, Scagnoli (2012) thinks a micro-lecture is a short recorded audio or video presentation on a single, tightly defined topic [10].

With the rise of new learning styles such as the flipped classroom, blended learning and mobile learning, the connotations of the micro-lecture change accordingly, and the micro-lecture is deemed as the one with videos as the main carriers, recording the brilliant teaching and learning process carried out by teachers for a certain knowledge point or teaching process in the teaching course of classroom education. Such a phase is not concerned with learning resources, instead, it concerns more about the process of the learning activity, and flipped classrooms [20], blended learning [21] and mobile learning [22] are also carried out as the major teaching approaches. The study in the forms above overturns the traditional mode characterized by teaching before learning, and allows students firstly to learn the micro-teaching resources by themselves before the class, make sufficient discussions in class and possibly realize the teaching process of learning before teaching. For example, Han (2019) [11] thinks the micro-lecture can adopt online or face-to-face mixed teaching methods, and intersperse various learning activities with a 3–5 min short speech, so as to strengthen the memory of the subject of the course, change students’ learning experience and break through the mindset of the course concept.

With the rise of various micro-lecture websites, the research on the connotations of micro-lecture enters the online course phase, the micro-lecture in this phase is defined as a micro-online video course with micro-videos as the core resource. The micro-lecture connotations of online courses are closely related to the rise of online learning websites; various online learning platforms break the barriers of the traditional classroom, make people study at any time and at any place and scholars think the micro-lecture is one kind of online study. For example, Yang and Tao (2015) [12] think micro-lectures are the instructional videos widely used in MOOCs. In this phase, scholars have reached a consensus and think the time of micro-lecture is no longer than 10 min, usually. For example, Wijaya and Weinhandl (2022) think the micro-lecture is a short, less than 10 min, video focusing on new knowledge and concept [23].

3. Definitions used in this paper

As the connotations, discussions and development of micro-lectures constantly deepen, micro-lecture research has gone through different development stages, but two points thereof are unchanged: (1) the micro-lecture is cored by micro-video [10,11,12,13,22]; (2) the time of the micro-lecture is shorter, and is generally less than 10 min [23]. Since this paper focuses on researching the use by teachers in universities of the micro-lecture, and exploring the use by teachers of the micro-lecture in the traditional teaching process and teaching reform, instead of the production of micro-online courses, the connotations of teaching processes are adopted. According to this paper, a micro-lecture is a minor course 10 min long or less, with a clear teaching objective and short contents, focusing on describing a topic, and its main embodiment approach is the video. Micro-lecture usage is that the teacher prepares micro-lecture in advance, uses in class or is used by a student in his/her preview or review of the class.

#### 2.1.2. Characteristics of Micro-Lecture

According to the existing research of scholars on micro-lectures, the micro-lecture has four characteristics, i.e., short time, conciseness, ease of share and attraction.

Presently, a consensus on micro-lecture study is that the micro-lecture has the characteristic of short time. The reason for the name of the micro-lecture is the short time of it [24]. The short time makes the micro-lecture unable to have the teaching time of approximately 45 min as same as that of classroom teaching of traditional higher education, but has the strengths unavailable in traditional classroom teaching. The short time can increase the attention of students, but the longer class time can make it difficult to keep the attention of students [25], so students may be distracted, scatterbrained or play on cell phones in class, and thereby their learning outcome may be affected. However, the characteristics of a short micro-lecture time decide that the micro-lecture is more suitable for the learning and cognition of the student, and allows the student to concentrate his/her attention in a limited time and thereby achieve the best learning outcome. From the perspective of short time, scholars studied the usage of micro-lectures and thought the short time could make students use their spare time to study, namely to study in the fragmented time when waiting for the bus, taking the subway or doing exercise, and then achieve the fragmented learning and mobile learning in the authentic sense; however, teachers use micro-lectures for teaching to meet the trend of fragmented learning and mobile learning, for example, Zhu and Zhu (2017) [26] thought the mobile, non-formal, fragmented, personal learning concepts started to threaten the traditional college education, so the design of micro-lectures must be added into teaching.

According to much research, the micro-lecture has the characteristic of conciseness; since the micro-lecture is teaching made in a short time, its time length decides the characteristic of conciseness [27]. The shortcoming of conciseness suggests that micro-lecture teaching cannot cover systematic and comprehensive knowledge points, has relatively single teaching objectives and additionally, has strengths that are unavailable in other teaching approaches. The conciseness makes the micro-lecture teaching have strong topicality. Every micro-lecture developed currently is for a certain knowledge point [20,28] and it includes difficult points in teaching and key teaching points [29], so its teaching theme is more outstanding. Some scholars, from the perspective of conciseness, studied the usage of micro-lectures. For students, a concise micro-lecture means convenient to use, since students can make their study concise and efficient; students can find the corresponding micro-lecture for their study in the case of needing to consolidate the learning outcome of certain knowledge points. For example, Scagnoli (2012) thinks “these short lectures encourage a self-directed model of learning, allowing students to select lessons to watch and to move through them at their own pace” [10]; however, concise micro-lectures demand more in the usage by teachers because it requires teachers to present the knowledge content to be taught accurately and concisely in a shorter time, for another example, Cao Jian-xia (2019), upon researching, thinks that teacher’s body language has a significant impact on student’s learning effect in the micro-lecture, stating knowledge points [24].

According to much research, the micro-lecture has the characteristic of share-easiness. Micro-lecture teaching is normally realized by a digitalized platform; thus, the electronic resources formed by micro-lecture therefrom can spread more quickly in a wider range at lower costs [30,31]. According to research, the micro-lecture teaching content developed by teachers can be made autonomous for learning repeatedly and several times by students via mobile devices [16]; for peer teachers, they can use the micro-lecture resources developed by other teachers to engage in their reference teaching. Students can also watch micro-lectures repeatedly, understand the knowledge points that are difficult to understand, revert to the learning outcomes to teachers and also make their interaction and communication with other students.

The research concludes that the micro-lecture is also attractive. Currently, micro-lecture teaching forms are majorly dynamic videos [32], with specific contents including PPT screen recording [27,33], animated video [34], teacher’s lecture [35], etc. Dynamic videos, relative to print media, are more attractive [36], and allow for post-editing and processing to add content enjoyment and accuracy, and thereby improve the attraction to students. Additionally, concise, short time, a strong theme and other characteristics of the micro-lecture can also increase the attraction to students. Some scholars compared the attractiveness of micro-lecture and other informationized education forms, for example, Wang et al. (2019) compared the idea that teachers use three forms of informationized education means, namely PowerPoint (PPT) skills, multimedia courseware production and micro lectures [13], where the results therefrom showed that the resources of the micro-lecture, as they are produced, can significantly improve the evaluation of teachers on information teaching.

By summarizing present research on the characteristics of micro-lecture, it can be seen that the present research focused on single characteristics, but did not focus on the impact of all of the characteristics of the usage by teachers using the quantitative method.

### 2.2. TAM and UTAUT

**Figure 1:**
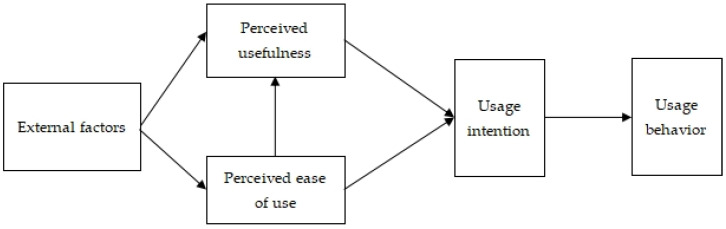
Technology Acceptance Model (TAM).

As modern information technology (IT) develops, more and more scholars start exploring what influences the will and behavior of people when accepting IT. Davis, firstly, put forward the Technology Acceptance Model (TAM) in 1989 [37], aiming to find out an effective behavior pattern to explain the behaviors of IT users when accepting new information systems. Such a model can be universally applied to explain and forecast the influence factors on the usage of IT. As shown in Figure 1, TAM verifies the influence of various variables, such as behavioral intention, behavioral attitude, perceived usefulness, perceived ease of use and external factors, on using behaviors of individuals.

Following this, additional scholars made verification and extension on TAM. In 2000, Venkatesh put forward TAM2 [38], and introduced the variable of the subjective norm in the TAM model; in 2003, Venkatesh put forward the Unified Theory of Acceptance and Use of Technology (UTAUT) [39], unified and refined four factors of performance expectancy, effort expectancy, social group influence and cooperation from the factors of TAM and introduced four control variables of sex, age, experience and voluntariness in the model.

In the application research of TAM and UTAUT, the discovery therefrom is that two technologies, namely risky and controversial technology and informationalized technology, were researched more. The former is that with technical application exists certain risks; the popularization of such controversial technology can only be made after winning the acceptance of the public, for example, Schmidthuber et al. (2020) used the TAM to study the acceptance of the public for mobile payment [40], and Wu (2014) [41] used the TAM to explore the acceptance of the public for transgenic technology. With the rise of the internet, some scholars used TAM to measure the acceptance degree of the public of informationalized technologies such as social media [42], e-commerce [43] and digital publishing [44]. The micro-lecture is also a product relying on IT, since the production of its teaching video, communication and use of its online video need IT; thus, certain scholars applied the TAM and UTAUT to the acceptance of micro-lecture, for example, Wijaya and Weinhandl (2022) [23] used the UTAUT model to explore “Factors Influencing Students’ Continuous Intentions for Using Micro-Lectures in the Post-COVID-19 Period”, the results therefrom suggested that effort expectancy (EE) and hedonic motivation (HM) had significant effects on attitudes, whose correlation with habit also influenced the continuous intention during this post-pandemic period. Wijaya et al. (2022) [45] used the UTAUT model to explore “Applying the UTAUT Model to Understand Factors Affecting Micro-Lecture Usage by Mathematics Teachers in China”, the results therefrom showed that BI (the behavioral intention) was positively affected by Performance Expectancy (PE), Effort Expectancy (EE) and Social Influence (SI). BI and facility conditions also had positive effects on user behavior; in contrast to other studies, SI had the most significant positive effect on BIs in our study.

Presently, research on TAM and UTAUT is lacking in the application in the crisis scenarios, and research on the TAM of the micro-lecture has already appeared, but less of them set the influence of the COVID-19 pandemic as a variable. In the context of the COVID-19 pandemic, the public’s mindset and behaviors may change accordingly; in the research herein, an attempt is made to introduce the influence variable of the COVID-19 pandemic, and provide new literature and a study case to TAM and UTAUT studies.

## 3. Materials and Methods

### 3.1. Research Variables and Measurable Variables

According to the characteristics of the micro-lecture, by combining the TAM and UTAUT models, and by selecting appropriate external factors and control variables to add to the research model, the factors influencing the university teachers’ intentions for using the micro-lecture in the post COVID-19 era were explored. In the TAM, external factors include system design characteristics, user characteristics, task characteristics, essence of development or execution process, training, policy impact, organizational structure, etc. In UTAUT, four factors of PE, EE, SI and cooperation affect the usage intention, and a simultaneous view is that sex, age, experience and voluntariness can play the role of adjustment. In this paper, the variable of micro-lecture characteristics was put forward according to the system design characteristics in TAM, the personal innovativeness variable was put forward according to the user characteristic in TAM, the teaching objective variable was put forward according to the task characteristic in TAM, the social relation variable was put forward according to the organizational structure in TAM and the SI in UTAUT, the policy impact variable was put forward according to the essence of development or execution process, training and policy impact in TAM and the PE, EE in UTAUT. According to the actual conditions of the COVID-19 pandemic and teachers in universities, by combining the control variables of sex, age, experience and voluntariness in UTAUT, six control variables of sex, age, title, discipline, use experience before the COVID-19 pandemic and use experience during the COVID-19 pandemic were proposed in this paper. The final research model includes 5 external factors, perceived usefulness, perceived ease of use, behavioral intention and 6 control variables. Thus, 8 research variables (a total of 24 measurable variables) and 6 control variables were obtained in the end.

#### 3.1.1. Micro-Lecture Characteristics

System design characteristics are one of the most important external factors of TAM, this paper researches the system characteristics of micro-lecture. According to the literature review, the micro-lecture is considered as one with various characteristics such as conciseness, short time, easy to share, attraction and capable of exerting influences on the usage behavioral intention of teachers in universities, see Table 3 for its connotations and measurable variables.

#### 3.1.2. Personal Innovativeness

User characteristics, among external factors of TAM, is the personality characteristic of how the user perceives new technology. An individual’s acceptance and attempt from teachers in universities to use new technology, a new form may affect his/her use of micro-lecture, therefore, the variable of personal innovativeness was put forward; additionally, measurable variables were put forward by referring to the scale measuring personal innovativeness, adopted by Kuo and Yen (2009) [46], when researching the acceptance behaviors of the innovative technology user, see Table 4.

#### 3.1.3. Teaching Objective

The task characteristic among the external factors of TAM refers to the objective to be achieved by using technology and the characteristic of such objectives. The task in the research in this paper is the teaching objective achieved by teachers in universities by using micro-lectures and the characteristics of such teaching objective, and then propose the variable of the teaching objective, according to the research of Yang (2018) [47], see Table 5.

#### 3.1.4. Social Relations

In TAM, the organizational institution in which the user is located is deemed to exert an influence on behaviors. In the theory of UTAUT, SI factors were also specially put forward, and they are defined as the degree that the important person thinks they should use new technology as perceived by the individual [39]. Teachers in universities stay mainly in schools and other teachers’ value concepts may be internalized by the teacher individual, unconsciously [48]; therefore, SI is deemed to exert influence on perceived usefulness. In this paper, the variable of SI is put forward to investigate the influence of SI, of where the individual is located, on the usage behavior of an individual. By referring to the SI variable in UTAUT, measurable variables were put forward, see Table 6.

#### 3.1.5. Policy Impact

Policy impact, one external factor mentioned in TAM refers to the influence exerted by the policy environment, formulated by the government, on the usage behaviors of the user; it is similar to PE and EE in UTAUT. This paper researches the variable of policy impact and investigates the influences of external policies such as material reward, fame reward and professional title appraisal on the micro-lecture teaching carried out by teachers in universities, see Table 7.

#### 3.1.6. Perceptibility and Behavioral Intention

In this paper, both usage intention and technology usage behavior in TAM and UTAUT are collectively unified into the behavioral intention, used for measuring whether teachers in universities use micro-lecture for teaching or not. However, two variables of perceived usefulness and perceived ease of use in TAM remained. In this paper, measurable variables were re-set by combining the background of the COVID-19 pandemic, see Table 8.

#### 3.1.7. Control Variables

According to the regulation theory, as proposed by the UTAUT model, sex, age and experience have a regulating function for usage intention [39]. According to the perspective of this paper, due to the particularity of teacher groups, technical titles [49] and the discipline [50] background of teachers, except for sex and age, among all population characteristics, may also affect their psychology and behaviors. The experience of teachers in universities in using micro-lecture can be divided into two types: one is the usage experience before the outbreak of the COVID-19 pandemic, and the other one is the usage experience during the period of the COVID-19 pandemic. The COVID-19 pandemic resulted in a significant increase in the popularizing rate of online education. By using such two variables, the one whether the COVID-19 pandemic improves the actual usage behavior of teachers for the micro-lecture or not can be effectively measured, but also the factor of the COVID-19 pandemic can be incorporated into the influence model of teachers’ micro-lecture usage intention as the variable. For the options of control variables, see Table 9.

### 3.2. Research Hypothesis and Research Model

#### 3.2.1. Influence Factors of Usage Intention

Usage intention refers to whether teachers in universities are willing to use micro-lectures for teaching. According to the TAM model and the UTAUT model, perceived usefulness, perceived ease of use and various control variables may exert influence on the usage intention of micro-lecture users; thus, Hypothesis H1a and H1b are, respectively put forward.

**Hypothesis** **(H1).** *Control variables of sex (a), age (b), discipline (c), technical title (d), usage experience before the outbreak of the COVID-19 pandemic (e) and usage experience in the COVID-19 pandemic (f) have a significant influence on the usage intention of teachers in universities for micro-lectures.*

**Hypothesis** **(H2).** *Perceived usefulness (a) and perceived ease of use (b) have a significant influence on the usage intention of teachers in universities for micro-lecture.*

#### 3.2.2. Influence Factors of Perceived Usefulness

According to the TAM model, various external factors and perceived ease of use exert significant influence on perceived usefulness, so the research hypothesis H3 is put forward.

**Hypothesis** **(H3).** *Social relations (a), policy impact (b), teaching objective (c), micro-lecture characteristics (d), personal innovativeness (e) and perceived ease of use (f) exert significant influence on the perceived usefulness of teachers in universities for micro-lecture usage.*

#### 3.2.3. Influence Factors of Perceived Ease of Use

In TAM, external factors may exert influence on the perceived ease of use. Therefore the research hypothesis H4 is put forward.

**Hypothesis** **(H4).** *Social relations (a), policy impact (b), teaching objective (c), micro-lecture characteristics (d) and personal innovativeness (e) exert significant influence on the perceived ease of use of teachers in universities for micro-lecture usage.*

#### 3.2.4. Research Model

**Figure 2:**
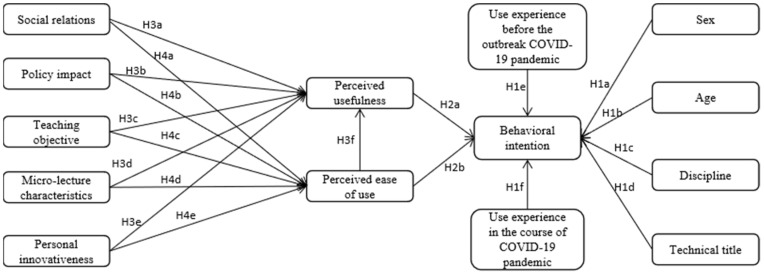
The research model of factors influencing university teachers’ intentions for using micro-lecture in the post COVID-19 era.

In this paper, according to the research hypotheses aforementioned, the research model of factors influencing university teachers’ intentions for using Micro-Lecture in the post COVID-19 era was put forward, see Figure 2 for the influence factors, influence path and research hypotheses.

### 3.3. Data Source and Reliability

#### 3.3.1. Release and Collection of Questionnaire

The questionnaire was designed as a Likert 5-level rating scale by using the research variables in Table 3, Table 4, Table 5, Table 6, Table 7 and Table 8, where the numbers from 1 to 5 were used to represent the options from strongly disagreeable to strongly agreeable. The control variables in Table 9 were designed as a single choice. In order to reflect the intention of teachers in universities sufficiently, the pattern of field survey and questionnaire was adopted. Accidental sampling is a non-probability sampling method adopted by an investigator at a special time and at a certain position of a specific community for cooperating research topic, aiming at selecting the answerer randomly. It is widely used in the investigation in which the specific entity is deemed as the respondent. In this paper, the objects are the groups in universities and are the comparatively particular groups, namely, the teachers who emerged in the teaching buildings in universities during the COVID-19 pandemic, they can represent the intention of the in-service teachers in the post COVID-19 era. Therefore, in this paper, the accidental sampling approach was adopted.

The investigation sites were the teaching buildings in 8 universities located along Xueyuan Road, Haidian District, Beijing City. The Xueyuan Road, Haidian District, Beijing City is one of the dense cluster districts of universities in Beijing City, where it is also the representative cluster area of universities. In the 1950s, due to the weak industrial base, China’s central government attached importance to the fostering of industrial construction talents, and so adjusted the schools and departments of universities, thus eight campuses of universities along Xueyuan Road were formed [51], such districts are the representative cluster area of universities in China. The questionnaires were released on the date of 1 November 2022, which was also the time when China recovered from COVID-19 and many universities resumed offline teaching.

Appendix A shows the number of full-time teachers in each university. Sampling is carried out according to the sampling ratio of 4%. The number of questionnaires issued and the number of questionnaires effectively recovered by each university are also shown in Appendix A. A total of 462 questionnaires were distributed, and 437 questionnaires were effectively recovered, with an effective recovery rate of 95%.

#### 3.3.2. Reliability and Validity

The Cronbach’s Alpha coefficient, proposed by Cronbach in 1951, was adopted to make a reliability analysis of questionnaire data. The reliability coefficient was 0.907 and the questionnaire reliability was above 0.9; additionally, 8 latent variables of every pattern were made in the reliability analysis, where the mean value therefrom was above 0.8, showing good reliability.

Test and measure the correctness of scale with validity analysis, adopt KMO statistical magnitude and Bartlett’s sphericity test to measure the structural validity. The purpose of structural validity is to analyze the explanation degree of 24 measurable variables in the model. The overall KMO test coefficient of questionnaire data was, respectively, 0.904, which met the demand of 0.7; the *p* value of Bartlett’s sphericity test was 0.000, signifying that questionnaire validity was good and the factor analysis could be made, the results of factor analysis indicated that all factors had an overall explanation of 65.552%. Therefore, the overall validity of the questionnaire was good.

#### 3.3.3. Analysis Method for Questionnaire Results

The structural equation model (SEM) is a statistical method used to analyze the relationship between variables based on their covariance matrix. It is an important tool for multivariate data analysis. If there is an interaction between independent variables, the regression model is not applicable, and SEM is more suitable for analysis. This study will use SEM to verify the influence factor model of teachers in universities for usage intention of micro-lecture in the post COVID-19 era.

The calculation formula between each measurable variable and exogenous latent variable is shown in Equation (1). Where *X*1 to *X*5 are exogenous latent variables, *X*1 refers to micro characteristics, *X*2 refers to personal innovation, *X*3 refers to teaching objectives, *X*4 refers to social relations and *X*5 refers to policy impact. z is the measurement error and α is the factor load of the measurable variable on the latent variable.

(1) $$\left[\begin{array}{c} a1\\ a2\\ a3\\ a4\\ b1\\ b2\\ b3\\ b4\\ c1\\ c2\\ d1\\ d2\\ d3\\ e1\\ e2\\ e3\\ e4 \end{array}\right]=\left[\begin{array}{ccccc} \alpha 1 & 0 & 0 & 0 & 0\\ \alpha 2 & 0 & 0 & 0 & 0\\ \alpha 3 & 0 & 0 & 0 & 0\\ \alpha 4 & 0 & 0 & 0 & 0\\ 0 & \alpha 5 & 0 & 0 & 0\\ 0 & \alpha 6 & 0 & 0 & 0\\ 0 & \alpha 7 & 0 & 0 & 0\\ 0 & \alpha 8 & 0 & 0 & 0\\ 0 & 0 & \alpha 9 & 0 & 0\\ 0 & 0 & \alpha 10 & 0 & 0\\ 0 & 0 & 0 & \alpha 11 & 0\\ 0 & 0 & 0 & \alpha 12 & 0\\ 0 & 0 & 0 & \alpha 13 & 0\\ 0 & 0 & 0 & 0 & \alpha 14\\ 0 & 0 & 0 & 0 & \alpha 15\\ 0 & 0 & 0 & 0 & \alpha 16\\ 0 & 0 & 0 & 0 & \alpha 17 \end{array}\right]\left[\begin{array}{c} X1\\ X2\\ X3\\ X4\\ X5 \end{array}\right]+\left[\begin{array}{c} z1\\ z2\\ z3\\ z4\\ z5\\ z6\\ z7\\ z8\\ z9\\ z10\\ z11\\ z12\\ z13\\ z14\\ z15\\ z16\\ z17 \end{array}\right]$$

The calculation formula between each measurable variable and endogenous latent variable is shown in Equation (2). *Y*1 to *Y*3 are endogenous latent variables. *Y*1 refers to perceived usefulness, *Y*2 refers to perceived ease of use and *Y*3 refers to behavioral intention. z is the measurement error and α is the factor load of the measurable variable on the latent variable.

(2) $$[\begin{array}{c} f1\\ f2\\ g1\\ g2\\ h1\\ h2\\ h3 \end{array}]= [\begin{array}{ccc} \alpha 18 & 0 & 0\\ \alpha 19 & 0 & 0\\ 0 & \alpha 20 & 0\\ 0 & \alpha 21 & 0\\ 0 & 0 & \alpha 22\\ 0 & 0 & \alpha 23\\ 0 & 0 & \alpha 24 \end{array}] [\begin{array}{c} Y1\\ Y2\\ Y3 \end{array}]+ [\begin{array}{c} z18\\ z19\\ z20\\ z21\\ z22\\ z23\\ z24 \end{array}]$$

The mathematical equation of the causal relationship among 3 endogenous latent variables, 5 exogenous latent variables and 6 control variables is shown in Equation (3). *n* is the residual term of the structural equation, reflecting the unexplained part of *Y* in the equation. γ is the influence of exogenous latent variables on endogenous latent variables. β is the relationship within the external latent variables. θ is the influence of control variables on exogenous latent variables.

(3) $$\left\{\begin{array}{c} Y1=\gamma_{11}X1+\gamma_{12}X2+\gamma_{13}X3+\gamma_{14}X4+\gamma_{15}X5+\beta_{21}Y2+n1\\ Y2=\gamma_{21}X1+\gamma_{22}X2+\gamma_{23}X3+\gamma_{24}X4+\gamma_{25}X5+n2\\ Y3=\beta_{31}Y1+\beta_{31}Y2+\theta_{1}I1+\theta_{2}I2+\theta_{3}I3+\theta_{4}I4+\theta_{5}I5+\theta_{6}I6+n3 \end{array}\right.$$

Amos 22.0 software is used to analyze the structural equation of the model and verify the proposed hypotheses.

## 4. Results

### 4.1. Descriptive Statistics of Variables

#### 4.1.1. Statistics of Research Variables

**Figure 3:**
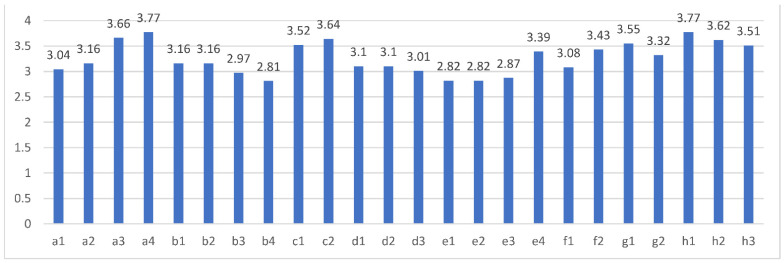
Mean values of measurable variables.

Figure 3 shows the mean values of 24 measurable variables. Among the variables of micro-lecture characteristics, the mean value of micro-lecture attraction (a4:3.77) was the highest, but the mean value of conciseness (a3:3.66), share-easiness (a2:3.16) and short time (a1:3.04) was declined, in turn. These results signified the high attraction of the micro-lecture for students is the most attractive reason for teacher usage. In variables about personal innovativeness, all respondents accepted new products and technology on a level that is not high(b1:3.16; b2:3.16; b3:2.97; b4:2.81). It might be related to the fact that teachers in universities were more traditional and conservative. Among the variables of teaching objectives, two measurable indicators were relatively higher, namely the diversified teaching topic (c1:3.52) and the abundant teaching form designs (c2:3.64). Among the variables of social relations, the mean values of d1(3.1) and d2(3.1) were the same and higher than d3(3.01). The results signified that behaviors of colleagues and important people have a higher influence on teachers’ micro-lecture usage. Among the variables of policy impact, the mean values of school requirement (e4:3.39) and help on technical title appraisal (e3:2.87) were both higher than those of material reward (e1:2.82) and fame reward (e2:2.82). The perceived usefulness, perceived ease of use and usage intention of teachers in universities for the micro-lecture were all relatively higher, where the mean values of those measurable variables were all above 3.

#### 4.1.2. Statistics of Control Variable

Table 10 shows the frequency of six control variables. Where: 169 teachers used micro-lectures before the outbreak of the COVID-19 pandemic (38.7%), 236 teachers used micro-lectures during the COVID-19 pandemic (54%). The COVID-19 pandemic improved the experience of respondents in using micro-lecture.

### 4.2. Results of SEM

#### 4.2.1. Correction and Fitting of SEM

The study in this paper adopted the AMOS software to make the structural equation analysis and verify the proposed hypothesis relations. The model was fitted by importing questionnaire data and by adopting the maximum likelihood estimate to analyze questionnaire data. On the basis of previous research [52], the study in this paper selected the absolute fit indicator, namely chi-square/freedom, RMSEA and relative fit indicators NFI, CFI and TLI, as the fitting standards; since the primary fitting standard of the questionnaire data was not good, the model was corrected by using the steps as follows.

(1) In the primary model, the path standardized coefficient of perceived ease of use and perceived usefulness for usage intention was higher than 1, signifying there was certain collinearity; moreover, the value *p* was higher than 0.05, namely the influence of perceived ease of use on usage intention is not significant; therefore, the path of perceived ease of use to usage intention was deleted.

(2) The model correction was finally made on the basis of the MI value. Through the modification indicators of AMOS software, the model correction indexes can be viewed. The one indicated by a double-headed arrow (<-->) shows the covariance correction index between residue variables, indicating the one that the model Chi-square value could be reduced at least if increasing a relative path between the residual variables of two measurable variables. A two-way path between two residuals with a large covariance correction index was added.

**Figure 4:**
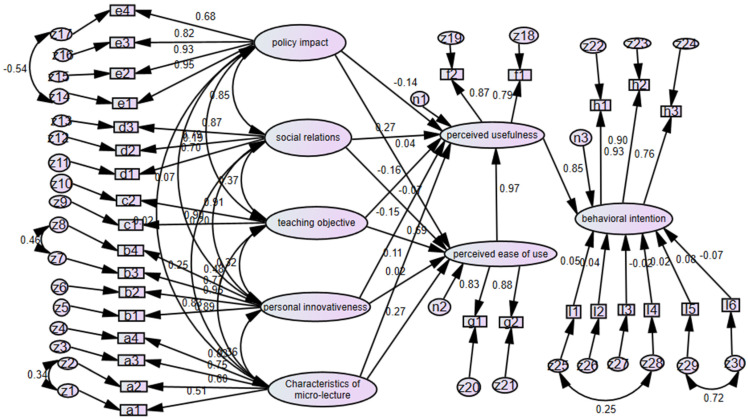
Validation of factors influencing university teachers’ intentions for using micro-lecture in the post COVID-19 era in AMOS.

After making the aforementioned correction, the data-fitting results could be obtained, see Table 11, where the degree of fitting of every indicator met the given requirements. For the indication of the fitted research model of factors influencing university teachers’ intentions for using Micro-Lecture in the post COVID-19 era, see Figure 4.

#### 4.2.2. Validation of Research Hypotheses

**Figure 5:**
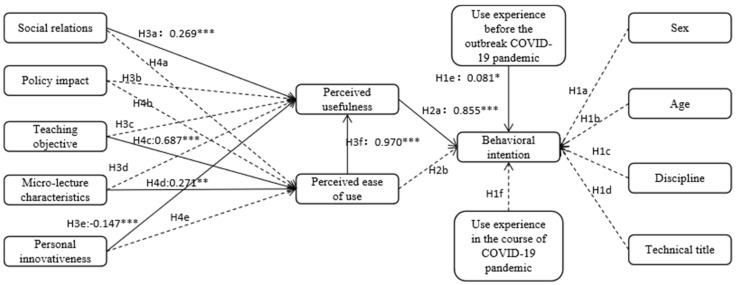
Validation of research hypotheses. (*: Significant at 0.1 level; **: Significant at 0.05 level; ***: Significant at 0.01 level).

Figure 5 shows the validation of the research hypotheses in the model. For the standardized coefficient of every path and the results of research hypotheses in the model, see Table 12. By validating the influences of control variables, it is clear that the usage experience before the outbreak of the COVID-19 pandemic had a significant influence on the behavioral intention of teachers in universities for micro-lecture usage, so H1e is true; other factors of sex, age, discipline, technical title and use experience in the course of COVID-19 had no significant influence on the usage intention, so H1a, H1b, H1c, H1d and H1f are not true.

In the validation of the influence of perceived ease of use and perceived usefulness on the usage intention of teachers in universities for micro-lecture, only the perceived usefulness (0.855 ***) exerted significant influence, so H2a was true; the path of perceived ease of use to behavioral intention was deleted in the first step of model modification, so H2b is not true.

Among the influence factors of perceived usefulness, social relations (0.269 ***), personal innovativeness (−0.147 ***) and perceived ease of use (0.970 ***) exerted significant influence, so H3a, H3e and H3f are accepted. However, policy impact, teaching objective and micro-lecture characteristics did not exert significant influence, so H3b, H3c and H3d are rejected. Among the influence factors of perceived usefulness, the influence weight of perceived ease of use was maximum, social relations came second and the influence weight of personal innovativeness was negative.

Among the influence factors of perceived ease of use, social relations, policy impact and personal innovativeness did not have significant influence, but teaching objective (0.687 ***) and micro-lecture characteristics (0.271 **) exerted significant influence, so H4c and H4d are true, but H4a, H4b and H4e are not true. Among the influence factors of perceived ease of use, the influence weight of teaching objectives was higher than that of micro-lecture characteristics.

## 5. Discussion

In the UTAUT model, sex, age and use experience had a regulating effect on usage intention and behavior [39]; subsequent related studies validated that the technical title and discipline might exert an influence on the psychology and behavior of teachers in universities [49,50]. However, a finding from the study in this paper is, in the scenarios of usage intention of teachers in universities for micro-lecture in the post COVID-19 era, only the micro-lecture use experience before the outbreak of the COVID-19 pandemic exerted influence on the usage intention, but sex, age, discipline, technical title and micro-lecture usage intention in the course of the COVID-19 pandemic did not exert influence on the usage intention of teachers in universities. The possible reason therein is the higher knowledge level of teachers in universities in Beijing, namely the typical middle class (bourgeoisie); their viewpoints and cognition for micro-lecture and other online education patterns are less affected by the demographic characteristics. Additionally, the experience of online education that had to be carried out in the course of the COVID-19 pandemic due to external factors did not affect the intention of teachers in universities for micro-lecture usage, only those groups contacted that used micro-lectures before the outbreak of the COVID-19 pandemic had a higher usage intention, it is related to the idea that the teacher groups in universities have a stationary self-viewpoint and they are not easy to be affected by external factors.

In the TAM model, perceived usefulness and perceived ease of use had a significant influence on the usage intention of users [37], but according to the finding from the study in this paper, in the scenario of the intention of teachers in universities for micro-lecture usage in the post COVID-19 era, only perceived usefulness exerted influence, perceived ease of use did not exert influence. It may be because those teacher groups were comparatively sensible and calm [53], only those teacher groups who feel that micro-lectures are authentically useful can increase their usage intention, but just the perception of ease of use cannot affect the final usage intention. The contribution of this finding is, if there is an eagerness to improve the micro-lecture usage intention of teachers in universities, it is a must to improve their cognition of the usefulness of micro-lecture, for example, make the production platform of micro-lectures quicker, and let the educational effect of micro-lecture be better.

Although in other research, policy impact might exert influence on perceived usefulness and perceived ease of use [37,39,46], it did not exert a significant influence on either perceived usefulness or perceived ease of use in this paper; this might be related to the psychology of teachers in universities as not easy to be affected by an external policy. This finding has four contributions: (1) social relations have a positive and significant impact on perceived usefulness, indicating that the interpersonal relationship network in which teachers in universities are located decides directly whether they feel the micro-lecture is useful or not, a good usage atmosphere that is only available in campus can make teachers in universities use micro-lectures better. (2) Personal innovativeness has a negative and significant impact on perceived usefulness, which indicates that teachers who like to try micro-lecture productions may find problems in them and will reduce their perception of the usefulness of micro-lectures; it indicates that the micro-lecture production platform should be more convenient and meet the requirements of college teachers. (3) Teaching objectives have a significant positive impact on perceived ease of use, when constructing micro-lecture production platforms, the one to facilitate and achieve teaching objectives should be adopted as the main direction. (4) Micro-lecture characteristics have a significant positive impact on perceived ease of use. The characteristics of micro-lectures, namely short time, conciseness, share-easiness and strong attraction, are the core elements of teachers in universities for selecting and using it, so the micro-lecture production platform must keep and develop all of these characteristics of micro-lectures as much as possible.

## 6. Conclusions

Firstly, the micro-lecture use experience before the outbreak of the COVID-19 pandemic has a significant influence on university teachers’ intentions for using micro-lectures. Secondly, perceived usefulness has an influence on university teachers’ intentions for using micro-lectures, while perceived ease of use does not. Finally, social relations and personal innovativeness have significant impacts on perceived usefulness, and teaching objectives and micro-lecture characteristics have significant impacts on perceived ease of use. 

There are still many deficiencies in the study in this paper. The study in this paper focused on the teachers in eight universities located along Xueyuan Road, Haidian District, Beijing City, they represent only the groups of teachers of a higher level in China, but the usage intention of other teachers in other universities was not widely explored, so the representativeness therein might be not enough. Therefore, it is important to expand the range of study in future so as to make it typical nationwide as much as possible.

## Figures and Tables

**Table 1:** Origin of the concept of micro-lecture.

Time	Institutions	Concepts Related to Micro-Lecture
1960	Affiliated school of the University of Iowa	It put forward the mini-course of a short-term course or course unit designed and executed against a certain theme, it is only a one- or two-hour class, and its content is relatively single and independent, most of the contents are developed on the basis of the common interests of students and teachers, and most of them focus on the depth of knowledge, instead of breadth of knowledge [7].
1960	Stanford University, USA	Dwight W.Allen, the director of its Teacher Training Department, proposed the micro-teaching method, which divides the complicated teaching process into multiple short processes, and divides the teaching contents into multiple single themes, knowledge particles or knowledge points [8].
1998	Singapore Ministry of Education	It offers the research project of Micro-Lessons, in which teachers are trained to construct the course for 30 min to 1 h, and the teaching objectives are single and concentrated, so it can provide teachers with a series of supports for making the specific teaching design [9].
2001	MIT	It works out the plan of Open Course Ware (OCW) for the execution of a mini video course, and offers the mini teaching video [16].
2004	England	It opens the teacher television channel, where every program video is 15 min. The program channel is widely accepted by teachers, and has accumulated the resources of mini-course videos that are 35 min [17].
2003	University of Pennsylvania	It offers an open class named “60-Second Lectures” [18].
2006	Harvard University	It attempts to promote the 8 min teaching video titled “The Inner Life of the Cell” [19]

**Table 2:** Development phase of micro-lecture connotation.

	Teaching Resource	Teaching Process	Online Course
**Forms**	Teaching video, courseware, materials, exercises	Teaching video, courseware, materials, exercises	Teaching video
**Usage**	Classroom teaching	Flipped classroom, blended learning and mobile learning	Online micro-lecture learning website
**Contents**	For a certain knowledge point, a certain teaching process	For a certain knowledge point, a difficult point	For a certain theme
**Time**	Shorter than the traditional class time	Shorter than the traditional class time	Within 10 min

**Table 3:** Connotations and measurable variables of micro-lecture characteristics.

Research Variable	Connotations	Measurable Variable
1. Characteristics of Micro-Lecture [10,25,27,36]	Measure the influence exerted by the native characteristics of micro-lecture on user.	a1 short time of a single micro-lecture may make me select and use it. a2 concise content of a single micro-lecture may make me select and use it. a3 share-easiness of a micro-lecture may make me select and use it. a4 strong attraction of a micro-lecture for the student may make me select and use it.

**Table 4:** Connotations and measurable variables of personal innovativeness.

Research Variable	Connotations	Measurable Variable
2. Personal innovativeness [46]	It refers to the intention that the user accepts a new product or new technology.	b1 I am curious about many things. b2 I think it is interesting to attempt new products, new technology. b3 I often search for information on new products, new technology. b4 I am usually a person who first attempts a new product among people of the same age.

**Table 5:** Connotations and measurable variables of course content.

Research Variable	Connotations	Measurable Variable
3. Teaching objective [47]	Influence of the teaching objective, which can be completed by using micro-lectures, on the usage of the user.	c1 using micro-lectures can make for diversified topic selection and content for teaching, which may make me select and use it. c2 using micro-lectures can make designs on abundant teaching forms, which may make me select and use it.

**Table 6:** Connotations and measurable variables of social relations.

Research Variable	Connotations	Measurable Variable
4. Social relations [39]	It refers to the influence of the social network in which an individual is located on the usage behavior of an individual.	d1 many colleagues around me use micro-lectures for teaching, which may make me select and use it. d2 the person who is important to me takes part in micro-lecture teaching, which may make me select and use it. d3 the person who is important to me thinks I should use micro-lectures for teaching, which may make me select and use it.

**Table 7:** Connotations and measurable variables of policy impact.

Research Variable	Connotations	Measurable Variable
5. Policy impact [37,39]	It refers to the influence of policy on the usage behavior of an individual.	e1 using micro-lectures for teaching gives a material reward, which may make me select and use it. e2 using micro-lectures for teaching gives a fame reward, which may make me select and use it. e3 using micro-lectures for teaching is helpful for professional title appraisal, which may make me select and use it. e4 the university requires me to add the application of micro-lecture, which may make me select and use it.

**Table 8:** Connotations and measurable variables of perceptibility and behavioral intention.

Research Variable	Connotations	Measurable Variable
6. Perceived usefulness [37]	Using micro-lectures for teaching can benefit teachers in universities.	f1 Free use of the micro-lecture software/platform in the period of the COVID-19 pandemic may make me prepare and make micro-lecture. f2 Easier use of the micro-lecture software/platform in the period of the COVID-19 pandemic may make me prepare and make micro-lecture.
7. Perceived ease of use [37]	The extent of the difficulty of teachers in universities in using micro-lecture teaching.	g1 Quickness of using the micro-lecture software/platform to make teaching content may make me use micro-lectures. g2 Micro-lectures can make me achieve better online education effects in the COVID-19 pandemic.
8. Behavioral intention [37,38,39]	Possibility of teachers in universities for teaching by using micro-lecture.	h1 In the post COVID-19 era, I am willing to use micro-lecture to teach. h2 In the post COVID-19 era, I am willing to recommend to other people to use micro-lectures to teach. h3 In the post COVID-19 era, I prefer to use micro-lectures to teach compared with other online teaching approaches.

**Table 9:** Control variable and their options.

Control Variable	Options
I1 Sex	male/female
I2 Age	23~32/33~42/43~52/53 or above
I3 Discipline	Science and engineering/non-science and engineering
I4 Technical title	Professor/associate professor/lecturer/teaching assistant
I5 Usage experience before the outbreak of the COVID-19 pandemic	Yes/No
I6 Usage experience in the COVID-19 pandemic	Yes/No

**Table 10:** Frequency statistics of control variables.

Sex	Age	Discipline	Title	Usage Experience before the COVID-19	Usage Experience in the COVID-19
Male (259)	23~32 years old (69)	Liberal arts (295)	Teaching assistant (51)	Yes (169)	Yes (236)
Female (178)	33~42 years old (181)	Non-liberal arts (142)	Lecturer (130)	No (268)	No (201)
	43~52 years old (116)		Associate professor (136)		
	53~62 years old (71)		Professor (120)		
N = 437	N = 437	N = 437	N = 437	N = 437	N = 437

**Table 11:** Fitted value for corrected data.

Index Description		Review Criterion	Actual Fitting Value
Absolute fitting index	Ratio of chi-square freedom	1–5	3.324
	RMSEA	Less than 0.05: signifying the good fitness; less than 0.08: acceptable.	0.073
	NFI	More than 0.9: signifying the good fitness; more than 0.8: acceptable.	0.863
Relative fitting index	CFI	More than 0.9: signifying the good fitness; more than 0.8: acceptable.	0.900
	TLI	More than 0.9: signifying the good fitness; more than 0.8: acceptable.	0.884

**Table 12:** Validation of path coefficient and research hypothesis.

Influence Factors		Affected Factors	Unstandardized Regression Weights	Standardized Regression Weights	S.E.	C.R.	*p*	Hypothesis	Results (Significant at 0.1 Level)
Sex	--->	Usage intention	0.107	0.048	0.07	1.516	0.129	H1a	Rejected
Age	--->	Usage intention	0.041	0.035	0.036	1.153	0.249	H1b	Rejected
Discipline	--->	Usage intention	−0.035	−0.015	0.071	−0.496	0.62	H1c	Rejected
Technical title	--->	Usage intention	0.023	0.02	0.035	0.644	0.519	H1d	Rejected
Use experience before the outbreak of COVID-19 pandemic	--->	Usage intention	0.18	0.081	0.099	1.812	0.07	H1e	Accepted
Use experience after the outbreak of COVID-19 pandemic	--->	Usage intention	−0.148	−0.068	0.097	−1.523	0.128	H1f	Rejected
Perceived usefulness	--->	Usage intention	0.968	0.855	0.053	18.262	0.000	H2a	Accepted
Perceived ease of use	--->	Usage intention	This path was deleted in the first step of model modification					H2b	Rejected
Social relations	--->	Perceived usefulness	0.301	0.269	0.108	2.782	0.005	H3a	Accepted
Policy impact	--->	Perceived usefulness	−0.108	−0.142	0.07	−1.557	0.119	H3b	Rejected
Teaching objective	--->	Perceived usefulness	−0.142	−0.155	0.161	−0.885	0.376	H3c	Rejected
Micro-lecture characteristics	--->	Perceived usefulness	0.17	0.114	0.201	0.844	0.399	H3d	Rejected
Personal innovativeness	--->	Perceived usefulness	−0.247	−0.147	0.065	−3.785	0.000	H3e	Accepted
Perceived ease of use	--->	Perceived usefulness	0.884	0.97	0.161	5.481	0.000	H3f	Accepted
Social relations	--->	Perceived ease of use	−0.087	−0.071	0.111	−0.784	0.433	H4a	Rejected
Policy impact	--->	Perceived ease of use	0.037	0.044	0.074	0.499	0.618	H4b	Rejected
Teaching objective	--->	Perceived ease of use	0.693	0.687	0.125	5.534	0.000	H4c	Accepted
Micro-lecture characteristics	--->	Perceived ease of use	0.443	0.271	0.213	2.078	0.038	H4d	Accepted
Personal innovativeness	--->	Perceived ease of use	0.028	0.015	0.065	0.426	0.670	H4e	Rejected

## Data Availability

The dataset generated and analyzed in this study is not publicly available; however, the dataset is available from the corresponding author upon reasonable request.

## Appendix A. Distribution and Recovery of Questionnaires

**Table A1:** Number of questionnaires issued and returned.

Universities	Number of Full-Time Teachers	Data Sources	Number of Questionnaires Issued	Number of Questionnaires Effectively Recovered
China Agricultural University	2007	http://www.cau.edu.cn/col/col10248/ (accessed on 1 September 2022)	85	80
Peking University Medical Department	715	https://www.bjmu.edu.cn/docs/2022-03/e470e704b9e54a678a531b4de9cff01e.pdf (accessed on 1 September 2022)	29	28
University of Science and Technology Beijing	1940	https://www.ustb.edu.cn/xxgk/xxjj/index.htm (accessed on 1 September 2022)	78	73
Beijing Language and Culture University	771	http://www.blcu.edu.cn/col/col15810/index.html (accessed on 1 September 2022)	31	26
China University of Mining and Technology, Beijing	710	https://www.cumtb.edu.cn/xxgk/xxgk.htm (accessed on 1 September 2022); Interview	28	27
Beihang University	2921	https://www.buaa.edu.cn/bhgk/jrbh.htm (accessed on 1 September 2022)	117	113
China University of Geosciences, Beijing	983	https://www.cugb.edu.cn/xxjj.jhtml (accessed on 1 September 2022)	39	37
Beijing Forestry University	1386	https://www.bjfu.edu.cn/2022/xxgk/xxjj/index.htm (accessed on 1 September 2022)	55	53
N			462	437
